# Performance of the Leicester risk assessment and Leicester practice risk scores for assessing the risk of undiagnosed type 2 diabetes or prediabetes in diverse populations: protocol for a systematic review of published validations and updates

**DOI:** 10.1186/s41512-026-00219-w

**Published:** 2026-01-15

**Authors:** Louise Haddon, Joie Ensor, Kamlesh Khunti, Laura J. Gray

**Affiliations:** 1 Department of Population Health Sciences, University of Leicester, Leicester, UK; 2 Department of Applied Health Sciences, School of Health Sciences, College of Medicine and Health, University of Birmingham, Birmingham, UK; 3 Birmingham Biomedical Research Centre, National Institute for Health and Care Research (NIHR), Birmingham, UK; 4 Diabetes Research Centre, University of Leicester, Leicester, UK; 5 NIHR Applied Research Collaboration East Midlands, University of Leicester, Leicester, UK; 6 NIHR Leicester Biomedical Research Centre, University of Leicester, Leicester, UK; 7 Leicester British Heart Foundation Centre of Research Excellence, University of Leicester, Leicester, UK

**Keywords:** Type 2 diabetes mellitus, Prediction, Prognosis, Systematic review, Risk, Study protocol, Diagnostic

## Abstract

**Background:**

Approximately one million adults in the UK are estimated to have undiagnosed type 2 diabetes mellitus (T2DM), with a further 5.1 million adults with nondiabetic hyperglycaemia (NDH) that does not meet the threshold for a diabetes diagnosis. The Leicester Risk Assessment score (LRA) and Leicester Practice Risk score (LPR) are diagnostic risk prediction models that estimate an individual’s risk of undiagnosed T2DM and NDH, developed for use in community and primary care settings respectively. The LRA is also used as a prognostic model; neither model has been updated since development. This study will systematically review all applications of these models as diagnostic and prognostic tools and any published updates to evaluate their performance in different populations. This review has been registered with PROSPERO (CRD420251005841).

**Methods:**

We will implement a citation search strategy to search Scopus, Web of Science and Google Scholar, restricted to full text, English language papers. Eligible papers will validate, update or modify either model. Data will be extracted using a form based on the Critical Appraisal and Data Extraction for Systematic Reviews of Prediction Modelling Studies (CHARMS) checklist; missing information will be sought from authors or estimated from other available information where possible. Meta-analysis of predictive performance measures will be completed if sufficient data exist. Subgroup and sensitivity analyses will be used to explore between-study heterogeneity and risk-of-bias impact.

**Discussion:**

This review will identify studies that have implemented, modified or validated the LRA and LPR for the risk of undiagnosed T2DM and NDH in different populations. This will allow summary measures, including level of uncertainty, of model performance to be calculated, making this highly relevant to individuals and stakeholders who recommend and implement these models. Review conclusions will also inform the potential update and recalibration of the models. This will ultimately lead to improved outcomes through earlier diagnosis and management.

**Supplementary Information:**

The online version contains supplementary material available at 10.1186/s41512-026-00219-w.

## Background

Approximately one million adults in the UK may have undiagnosed diabetes [1]. Left untreated, Type 2 diabetes (T2DM) can cause serious complications that place a significant burden on the individual, with reported life expectancy up to ten years less than individuals without diabetes [2]. There is also significant burden on the wider health economy, with approximately 10% of annual NHS expenditure spent on T2DM [3]. Non-diabetic hyperglycaemia (NDH) and prediabetes are terms used to describe raised blood glucose levels which do not meet the criteria for diagnosis of T2DM [4]. There are a further 5.1 million adults in the UK within this group [1] and it is estimated that between 5 and 10% will go on to develop T2DM [5–7]. It is believed that up to 50% of T2DM diagnoses can be prevented or delayed [8]. Where individuals do not progress to a diabetes diagnosis, NDH is still associated with an elevated long-term risk of complications including cardiovascular and renal disease [9].

The Leicester Diabetes Risk Assessment score (LRA) [10] was developed over 15 years using a diagnostic prediction model to identify undiagnosed T2DM and prevalent NDH. The model was originally used as a ‘tick box’ questionnaire where each answer generated a numerical score. The total score is then used to assess the risk of having undiagnosed T2DM or prevalent NDH. Included predictors are age, sex, ethnicity, first degree family history of T2DM, waist circumference, body mass index (BMI) and antihypertensive medication or hypertension.

The LRA is recommended by the National Institute for Health and Care Excellence (NICE) as part of a two-stage strategy to identify adults at high risk of T2DM [11]. The Leicester Practice Risk score (LPR) is similar to the LRA but was developed for use as a diagnostic prediction model within a primary care setting, therefore waist circumference was not included as this is poorly recorded in primary care records [12].

Clinical prediction models can be broadly split into two types: diagnostic models estimate an individual’s risk (or probability) of a specific health condition currently being present, whilst prognostic models estimate the risk of developing a specific health outcome over a specified time-period [13]. Although diagnostic and prognostic models share some characteristics, they are developed for different purposes. It is possible that a well performing diagnostic model can be a poor prognostic model if included predictors do not accurately capture the temporal relationship between the predictors and outcome of interest. Despite being developed using diagnostic prediction models to identify individuals who currently have undiagnosed T2DM or NDH, the LRA and LPR have has subsequently been used to identify individuals who may go on to develop T2DM [14, 15].

Populations and care pathways can change over time, therefore the performance of prediction models may also change and become less accurate [16]. The LRA/LPR have been evaluated in different populations as both a diagnostic and prognostic models and modifications have been suggested. This review will be the first to bring together all external validations of the models in different populations, including the addition of any new predictor variables. Thus, this review is important as it will yield a summary measure of both model’s discrimination and calibration in a wider population, including the level of uncertainty surrounding these, making this highly relevant to individuals and stakeholders who recommend and implement the models. This review will also inform an update and recalibration of the models.

### Aims and objectives

This review aims to (a) assess the performance of the LRA and LPR, in terms of discrimination, calibration and clinical utility, to predict an individual’s risk of undiagnosed T2DM and/or NDH by summarising available data in a meta-analysis to assess model performance, (b) assess the effect of any additional predictor variables added to either model, including the strength of association for existing predictor variables and (c) performance in predicting prevalent (diagnostic) as well as future disease (prognostic) will be assessed. Gray et al. (2010) did not publish the LRA model intercept in the original paper; the authors have provided this on request. Therefore, the review will also consider whether the full model was validated, or the validation estimated the model intercept, essentially recalibrating the model, when assessing performance in new populations.

## Methods

The protocol is registered in PROSPERO (CRD420251005841) and reporting follows the Preferred Reporting Items for Systematic Reviews and Meta-Analyses Protocols (PRISMA-P) 2015 statement [17]. Reporting of this review will adhere to Transparent reporting of multivariable prediction models for individual prognosis or diagnosis: checklist for systematic reviews and meta-analyses **(**TRIPOD-SRMA) guidelines [18], and the guidelines provided by the Cochrane Prognosis Methods Group [19].

The review question has been outlined in Table 1, according to the PICOTS format [20]. To achieve these aims, we will conduct a systematic review to identify external validation studies published since August 2010, where the LRA or LPR has been implemented, with or without updating, and model impact studies that applied the LRA model and/or the LPR, or any modified version of, that was originally developed by Gray et al. (2010) and Gray et al. (2012). We will include all studies assessing the performance of the model, whether the primary objective of the study was to validate the LRA/LPR or to use the LRA/LPR as a comparator for other prognostic models.

**Table 1:** Review question in PICOTS format

Characteristics	Details of what will be considered
1. Population	Individuals without a confirmed diagnosis of T2DM and no confirmed diagnosis of non-diabetic hyperglycaemia/prediabetes
2. Index prediction model(s)	Leicester Diabetes Risk score (LRA) published by Gray et al. 2010 Leicester Practice Risk score (LPR) published by Gray et al. 2012
3. Comparator prediction model(s)	Comparing predictive accuracy of all applications of LRA or LRP, including those updated with additional predictors and/or recalibrated
4. Outcomes	Any reported performance measures of the models, including discrimination and calibration
5. Timing (two elements)	Baseline: no T2DM or persistent hyperglycaemia diagnosis Outcomes: Diagnostic: risk of having either undiagnosed T2DM or NDH Prognostic: developing T2DM over stated time period
6. Setting	To fully assess model performance in all populations it has been implemented, all studies regardless of care setting and geographical location will be included.

### Study eligibility criteria

Studies will be eligible for inclusion in this review if they meet the following criteria: full-text publications in English, assessment of the performance of the LRA or LPR including diagnostic, calibration, discrimination and cost utility performance measures. Studies available in abstract form only will not be included as it is unlikely that enough information to adequately describe the target population and assess study quality and model performance would be included.

Studies involving participants with an existing T2DM or NDH diagnosis will be excluded. No restrictions will be placed on the target population in order to fully assess model performance in all settings.

### Search strategy

A forwards citation search strategy focused on the LRA and LPR will be used. Key publications will be identified as the primary papers (seed references) a priori. Titles and abstracts of published papers that have cited any of the key publications will be collected; relevant retrieved articles will then be used as new key references for a further iteration of citation searching. The process will repeat until no further eligible studies are identified [21]. The number of iterations will be reported.

It is acknowledged that citation searching is only an effective strategy if all eligible studies cite relevant early work [22]. Key references will include the original model development papers and three subsequent validations of one or both models completed by or with the original authors to maximise the likelihood of finding all relevant papers, included in appendix 1.

For each iteration of citation search, abstracts will be screened and grouped by model into one of three categories by two independent reviewers: papers that validate or implement the original clinical prediction model; papers that modify/amend the original prediction model (e.g. by amending or adding predictor variables); papers that are not relevant to the focus of this review.

### Information sources

Citations and references from the key publications will be identified using the following databases to maximise the possible number of citations:

- Scopus.
- Web of Science.
- Google Scholar.

How the model is described (name and keywords) within included papers will be extracted and then a keyword search in the PubMed database using these key descriptors will be performed to ensure the search strategy is as comprehensive as possible. In addition to the electronic searches, reference and citation lists of the retrieved articles will be hand-searched for further papers of interest.

### Data management

Screening will be performed using Covidence systematic review software [23] and selected articles, including PDF files, will be managed using Zotero [24].

### Selection process

Two reviewers will independently screen titles and abstracts for eligibility, followed by full text assessment. Disagreements will be resolved by discussion; in cases of no consensus, final resolution will be achieved by involving a third reviewer. Study selection will be documented in a flow chart (PRISMA) [17].

### Data collection process

Data will be extracted independently by two reviewers, in a piloted data extraction form based on the Critical Appraisal and Data Extraction for Systematic Reviews of Prediction Modelling Studies **(**CHARMS) checklist [17, 25]; the applicability and appropriateness of the extraction form will be tested using the first five included studies. Any disagreements that cannot be resolved by discussion will be referred to a third reviewer.

### Data items

Data extraction will include:

- Study information: author, title, publication date, date of validation, source, country.
- Source of data: e.g. registry data, existing cohort.
- Which model is being assessed (LRA, LPR or both, whether full model including intercept is used).
- Study design characteristics (prospective or retrospective, type).
- Patient characteristics including mean age, % male, family history of diabetes, mean BMI, % history of hypertension, ethnicity.
- Predictors included in the model including continuous or categorised, methods of measurement, thresholds for continuous predictors.
- Completeness of data (missing data, losses to follow up) and how missing data are dealt with (complete case or type of imputation).
- Model validation: total sample size, total number of events, target population, setting, whether model was recalibrated, predictor effects adjusted.
- Outcome measures used for diagnosis including diagnostic code of T2DM or NDH from routinely collected data, HbA1c, fasting plasma glucose, random plasma glucose, oral glucose tolerance test and cut-off points used.
- Timespan of prediction will be recorded to assess how the model is being used in practice and whether the model is used as a diagnostic or prognostic tool.
- Model performance measures, including validation performance statistics for calibration, discrimination and overall performance measures including 95% confidence intervals or standard errors.
- For calibration: calibration slope, intercepts, goodness-of-fit test or observed/expected (O/E) outcome ratios if reported. The number of observed events and the number of expected events will be extracted or derived from plots. As a binary outcome, Brier score will be extracted if available. Calibration plots (curve, slope) will be assessed and strength of calibration methods used will be rated [26, 27].
- For discrimination: the c-statistic, including uncertainty measures (confidence interval or standard error.). As the c-statistic is identical to the area under the receiver operating curve (AUROC), this will also be used.
- Mean values and standard deviations of the LRA, LPR and their predictors will be extracted to explore the effect of patient characteristics / case-mix on LRA/LPR performance.
- Sensitivity and specificity (also reported as true positive fraction and false positive fraction respectively), defined as the probability of correct classification in individuals with and without the target condition, are used to assess accuracy of a diagnostic test and are recommended for meta-analysis [28].
- Positive and negative predictive values, the probability of the health outcome given a positive test result and the probability of not having the health outcome given a negative test result for threshold values are described as more interpretable and clinically relevant than sensitivity and specificity [28].
- The distribution of the linear predictor to determine how different the study sample is from the original develop sample (this can inform how we interpret the performance at validation).
- Statistical methods used, including if/how any new predictors were included and any recalibration, how missing data was handled.
- Any clinical utility or cost effectiveness measures reported e.g. net benefit, decision curve analysis, incremental cost-effectiveness ratios.

### Missing data

If performance measures are not explicitly stated, methods outlined by Debray et al. (2017 and 2018) [20, 29] will be used to estimate these; missing performance measures and confidence intervals or standard errors will be calculated if the required information is reported. Missing total O: E ratio and standard error will be estimated using statistical equations using the total number of observed and expected events and the total sample size. Calibration-in-the-large will be estimated using the linear predictor and regression co-efficients of the model. A sub analysis will be undertaken for studies following appropriate quality standards, such as TRIPOD guidelines.

Variance or standard deviation of key patient characteristics can be estimated from reported ranges or histograms. Missing positive and negative predictive values will be generated from sensitivity and specificity [28]. Where relevant information is not reported, efforts will be made to contact the study authors to request this. It is possible that some applications of the model may include additional predictor variables or exclude some key parameters. The effect of this on model performance will be examined in subgroup and sensitivity analyses.

Estimates will be obtained using methods implemented in Stata package [30]. If five or more studies are available, the same package will be used to meta-analyse model performance measures. If meta-analysis is not possible, results will be presented as a narrative.

### Risk-of-bias assessment

Risk of bias of included studies will be assessed using the PROBAST (Prediction study of Risk of Bias Assessment Tool) [31] by two independent reviewers according to the following PROBAST domains: participants, predictors, outcome, analysis. Based on answers to the signalling questions within each domain, studies will be rated as low, high or unclear risk of bias. If information required to assess risk of bias is missing, study authors will be contacted to request additional information.

### Assessment of heterogeneity

Possible causes of heterogeneity may be differences in study characteristic or quality, difference in baseline risk of the prevalence of T2DM across different populations, performance estimates may be based on validations of a modified model with adjusted parameters [29] or if a predictor variable included in the model is not measured or not included in one or more studies (systematically missing predictor variables) [32]. It is possible that proxy predictor variables may have been used in some studies, for example if waist circumference is not recorded.

### Assessment of reporting deficiencies

Whilst it is recommended that all prediction models report calibration and discrimination, it has been reported that calibration and discrimination measures are often poorly reported or not reported at all [33]. Therefore, this review will evaluate reporting deficiencies in included studies. Funnel plots will be used to visually assess for any evidence of publication bias [34].

### Data analysis and synthesis

All following analyses will be performed for the LRA and LPR separately so that each individual model is evaluated.

Performance measures will be summarized in tables and illustrated graphically. As the LRA and LPR are diagnostic models, they can be evaluated following methods described for meta-analysis of accuracy of a diagnostic test for use in NICE decision models. This uses a Bayesian approach, allowing more complex analyses considering multiple thresholds i.e. specific thresholds for risk grouping, fitting a random effects model for sensitivity and specificity [28].

A random-effects meta-analysis of calibration (O: E ratio, calibration slope and calibration intercept) and discrimination (c-statistic or AUROC) [28, 35] will be performed if more than five studies are sufficiently similar [36]. The c-statistics will be transformed with a logit transformation of the c-statistic, using statistical package Stata [30]. If performance measures are not reported, they will be estimated according to methods outlined by Debray et al. [20, 29]. It is acknowledged that many validations may not have used the original LRA model intercept as this was not included in the original paper.

Where appropriate, meta-analyses will be performed using a random-effects model to account for heterogeneity; between-study variance ($\:{\tau\:}^{2}$) and the percentage total variation in the study estimate due to between-study heterogeneity ($\:{I}^{2}$) will be calculated. An approximate 95% prediction interval for discrimination (C-statistic) and calibration (O: E ratio) measures will be calculated [20].

Although no Grading of Recommendations, Assessment, Development, and Evaluations (GRADE) guidance currently exists for rating certainty of prediction model performance, a GRADE approach to assessing certainty in model calibration (assessed by the O: E ratio) [37] and proposing different methods to choose a threshold for rating certainty of evidence when assessing discrimination [38] has recently been published.

### Subgroup analysis and investigation of heterogeneity

Meta-regression and subgroup analyses will be used to evaluate model performance if the number of identified studies supports this. Foroutan et al. (2021) [37] suggest reporting calibration separately for different risk categories or clinically meaningful subgroups as an overall O: E ratio may appear adequate despite a model underestimating the risk in half of the population and overestimating the risk in the other half.

To identify potential sources of heterogeneity, we plan to compare the model performance in the whole study population with that in subgroups of studies that included participants with the following characteristics:

- By country.
- By method/criteria used to diagnose diabetes.
- Validation based on score or model.

It is expected that most studies will report aggregate data. Meta regression to investigate the association between patient-level characteristics and intervention effect can lead to ecological bias when using aggregate data [39, 40] and so characteristics in the target populations will be summarised and explored in subgroup analyses if little overlap exists, unless individual patient-level data is available.

### Sensitivity analysis

Sensitivity analysis will be used to test the effects of:

- estimation of any missing performance measures, if this method is used.
- risk-of-bias rating, if a sufficient number of studies within each rating is found.
- effect of estimating 95% prediction intervals and 95% confidence intervals on levels of certainty by exploring any changes between reported and estimated.

## Discussion

The results of this review will identify all studies that have implemented, modified or validated the LRA and LPR as diagnostic models estimating the risk of having undiagnosed T2DM and/or NDH in different populations. This will allow summary measures, including level of uncertainty, of model performance to be calculated, making this highly relevant to individuals and stakeholders who recommend and implement these models. The conclusions of the review will also inform the potential update and recalibration of the models. As the LRA is now increasingly used as a web-based tool, it provides the opportunity for a more complex model that can still be completed by a lay person. This will ultimately lead to improved outcomes for those with undiagnosed T2DM and prediabetes through earlier diagnosis and management.

NICE guidelines (PH38) for the prevention of T2DM in people at high-risk list three examples of risk assessment tools: the LRA, LRP and QDiabetes risk calculator [11]. This review focuses on the LRA and LRP models as these were developed for use in the UK to identify undiagnosed (prevalent) T2DM and/or NDH, allowing interventions such as the NHS Diabetes Prevention Programme to be targeted to individuals at high-risk. QDiabetes is a prognostic model that identifies the (incident) risk of developing diabetes over the next 10 years but has been developed for use by health professionals, explaining the decision not to include this model in the review [41, 42].

As this review focuses on implementation and validation of two specific clinical prediction models, the research team deemed there to be a high probability that the original papers describing the model developments would be referenced in relevant studies. Therefore, it is expected that a high percentage of titles from the searches will be included, given the citation search strategy suggested. Wright et al. (2014) [43] compared the performance of database search strategies and citation search strategies and found that using this combination of resources stated in this protocol could identify 99.7% of total citations found by a traditional database search.

Any review of clinical prediction models is limited by how models are reported. The original paper for the LRA did not report the full model, with no intercept included, although the corresponding author provides this on request. Validations that estimate the model intercept could be considered recalibration and this will need to be considered when evaluating reported performance measures. The LRA has been used as a diagnostic and prognostic tool, despite being developed as a diagnostic model using a cross-sectional dataset. This may result in separate analyses to evaluate differing model performance when predicting prevalent and incident T2DM.

Any future amendments to this protocol that result from knowledge acquired during the pilot data extraction stage or during initial iterations of citation searches will be documented in a separate section with justification for any changes included.

## Supplementary information

Supplementary Material 1. Appendix 2: PRISMA-P checklist

## Data Availability

All data generated from this review will be available from the corresponding author on reasonable request.

## Appendix 1

Draft search strategy.

The papers that will be used as key primary papers are:

- Gray LJ et al. (2010). The Leicester Risk Assessment score for detecting undiagnosed Type 2 diabetes and impaired glucose regulation for use in a multiethnic UK setting.
- Gray LJ et al. (2012). Detection of impaired glucose regulation and/or type 2 diabetes mellitus, using primary care electronic data, in a multiethnic UK community setting.
- Gray LJ et al. (2012). Implementation of the automated Leicester Practice Risk Score in two diabetes prevention trials provides a high yield of people with abnormal glucose tolerance.
- Gray LJ et al. (2014). External validation of two diabetes risk scores in a young UK South Asian population.
- Barber SR et al. (2017). External national validation of the Leicester Self-Assessment score for Type 2 diabetes using data from the English Longitudinal Study of Ageing.

Relevant retrieved articles will then be used as new key references for a further iteration of citation searching. The process will repeat until no further eligible studies are identified. The number of iterations will be reported.

Model description (name and keywords) within included papers will be extracted and then a keyword search in the PubMed database using these key descriptors will be performed.

## Abbreviations

T2DM: Type 2 diabetes
LRA: Leicester Risk Assessment score
LRP: Leicester Practice Risk Score
BMI: body mass index
NICE: National Institute for Health and Care Excellence
PICOTS: Population Index Prediction Model, Comparator, Outcomes, Timing, Setting
PRISMA-P: Preferred Reporting Items for Systematic Reviews and Meta-Analyses Protocols
CHARMS: Critical Appraisal and Data Extraction for Systematic Reviews of Prediction Modelling Studies
TRIPOD-SRMA: Transparent reporting of multivariable prediction models for individual prognosis or diagnosis: checklist for systematic reviews and meta-analyses
AUROC: Area under receiver operating curve
PROBAST: Prediction study of Risk Of Bias Assessment Tool
GRADE: Grading of Recommendations, Assessment, Development, and Evaluations
